# New Insight into the Antifibrotic Effects of Praziquantel on Mice in Infection with *Schistosoma japonicum*

**DOI:** 10.1371/journal.pone.0020247

**Published:** 2011-05-24

**Authors:** Yue-Jin Liang, Jie Luo, Quan Yuan, Dan Zheng, Ya-Ping Liu, Lei Shi, Ying Zhou, Ai-Ling Chen, Yong-Ya Ren, Ke-Yi Sun, Yan Sun, Yong Wang, Zhao-Song Zhang

**Affiliations:** 1 Department of Pathogen Biology, Key Laboratory of Pathogen Biology of Jiangsu Province, Nanjing Medical University, Nanjing, Jiangsu, China; 2 Department of Pathology, Nanjing Medical University, Nanjing, Jiangsu, China; 3 Department of Microbiology and Immunology, Nanjing Medical University, Nanjing, Jiangsu, China; The George Washington University Medical Center, United States of America

## Abstract

**Background:**

Schistosomiasis is a parasitic disease infecting more than 200 million people in the world. Although chemotherapy targeting on killing schistosomes is one of the main strategies in the disease control, there are few effective ways of dealing with liver fibrosis caused by the parasite infection in the chronic and advanced stages of schistosomiasis. For this reason, new strategies and prospective drugs, which exert antifibrotic effects, are urgently required.

**Methods and Findings:**

The antifibrotic effects of praziquantel were assessed in the murine models of schistosomiasis japonica. Murine fibrosis models were established by cutaneous infection with 14±2 *Schistosoma japonicum* cercariae. Then, the mice of both chronic (8 weeks post-infection) and advanced (15 weeks post-infection) schistosomiasis were treated by gavage of praziquantel (250 mg/kg, once daily for 3 days) to eliminate worms, and followed by praziquantel anti-fibrosis treatment (300 mg/kg, twice daily for 30 days). The fibrosis-related parameters assessed were areas of collagen deposition, content of hydroxyproline and mRNA expressions of Col1α1, Col3α1, α-SMA, TGF-β, MMP9, TIMP1, IL-4, IL-10, IL-13 and IFN-γ of liver. Spleen weight index, alanine aminotransferase activity and liver portal venous pressure were also measured. The results showed that anti-fibrosis treatment improved liver fibrosis, splenomegaly, hepatic function, as well as liver portal hypertension. In order to confirm the anti-fibrotic properties of praziquantel, we established a CCL_4_-induced model and revealed that CCL_4_-induced liver fibrosis was inhibited by PZQ treatment for 30 days. Furthermore, we analyzed the effects of praziquantel on mouse primary hepatic stellate cells (HSCs). It is indicated that mRNA expressions of Col1α1, Col3α1, α-SMA, TGF-β, MMP9 and TIMP1 of HSCs were all inhibited after praziquantel anti-parasite treatments.

**Conclusions:**

The significant amelioration of hepatic fibrosis by praziquantel treatment validates it as a promising drug of anti-fibrosis and offers potential of a new chemotherapy for hepatic fibrosis resulting from schistosomiasis.

## Introduction

Schistosomiasis is a tropical parasitic disease caused by blood-dwelling fluke, schistosome. According to WHO, 200 million people are infected by the parasite worldwide, leading to the loss of 1.53 million disability-adjusted life years [1]. The main pathologic lesions of hepatic schistosomiasis are the granuloma formation around schistosome eggs at acute stage of the infection, followed by the liver fibrosis at chronic and advanced stages [2]. Hepatic fibrosis of schistosomiasis results from a massive deposition of extracellular matrix (ECM) in the periportal spaces, leading to blockage of the portal veins, portal hypertension, splenomegaly, portocaval shunting, and gastrointestinal varices [1]. The persistent fibrosis of chronic schistosomiasis may cause hepatic cirrhosis and high mortality rate for liver cancer [3]. Although chemotherapy eliminates matured worms effectively and prevents the accumulation of schistosome eggs, less effective durgs are direct to reverse the existing hepatic fibrosis, especially at chronic and advanced stages of schistosomiasis. Therefore, treatment targeting hepatic fibrosis of schistosomiasis remains a challenging proposition [4].

Hepatic stellate cells(HSCs) are considered as an important liver resident cells, which are the major cellular sources of extracellular matrix (ECM) and play a key role in the process of hepatic fibrosis [5], [6]. Deposition of collagen, imbalance of matrix metalloproteinase (MMP)/tissue inhibitor of metalloproteinase(TIMP) and secretion of profibrotic cytokines caused by activated HSCs are described previously in schistosomiasis and other infectious diseases [7], [8]. According to their pivotal role, HSCs have been recognized as the therapeutic targets of hepatic fibrosis caused by permanent alcohol abuse [9], nonalcoholic steatohepatitis [10], Hepatitis C [11] and schistosomiasis [12].

Praziquantel (PZQ), as a safe anti-schistosome drug, has been used for more than 30 years [13]. It has been documented that PZQ has only mild side-effect and very low toxicity in animals [14]. No important long term safety problems have been observed in people and the safety is also considered in both children and pregnant women [15]. Reversal of schistosome-induced pathology after treatment of PZQ has been described. Parasitological cure of murine *Schistosoma monsoni* infection was followed by eventual partial reversal of liver fibrosis [16]. Researchers showed that patients in Ethiopia and Uganda had improvement or resolution of schistosomal periportal thickening/fibrosis after parasitologic cure of PZQ [17], [18]. The main explanations of these results are presumed to be probably a removal of schistosomal worms and subsequent reduction of egg deposition. However, the exact mechanism is not clear.

Recent years, in addition to anti-helminth effect, anti-inflammation of PZQ has been reported [19]. Report also showed that eight- weeks- continuing treatment with PZQ could inhibit the development of pulmonary granulomas caused by vein injection of *Schistosoma japonicum* (*S. japonicum*) eggs, suggesting that PZQ may have the effect on fibrosis [20]. It seems that PZQ may not only eliminate the parasites, but also influence the immune response of the host, and consequently exerts dual effects on the infection. The aim of our study is to investigate whether PZQ has the direct therapeutic action on liver fibrosis of schistosomiasis japonica and prospect it as a well tolerated, easily administered and inexpensive drug for new treatment strategy of hepatic schistosomiasis.

## Materials and Methods

### Animals and drug

Female BABL/c mice (Comparative Medicine Center of Yangzhou University, Yangzhou, China), 6–8 weeks old, were used throughout the study and maintained according to guidelines approved by the Nanjing Medical University Animal Experiment and Care Committee. *S. japonicum* cercariae of chinese mainland strain were obtained from the infected *Oncomelania hupensis* (Jiangsu Institute for Schistosomiasis Control, Wuxi, China). PZQ was purchased from Sigma and resuspanded in 1% carboxymethyl cellulose carrier for use.

### Establishment of infection model and strategy of PZQ treatment

The BABL/c mice were infected cutaneously with 14±2 *S. japonicum* cercariae [21].The liver fibrosis models were established after the mice being infected for 8 weeks and 15 weeks, respectively. Intragastric administrations of PZQ (250 mg/kg/24 hours) for 3 days and continuing 1% carboxymethyl cellulose carrier up to 30 days were carried out as an anti-parasite intervention [16]. Intragastric administrations of PZQ (300 mg/kg/12 hours) for one month were carried out as an anti-fibrosis intervention. Both interventions were carried out for the mice of eight- weeks-infection and fifteen- weeks-infection, respectively (n = 10 in each group). A low dose administration of PZQ (75 mg/kg/12 hours) for one month was also used for the mice of fifteen- weeks-infection (n = 10). Concurrent control of untreated mice (n = 10) with liver fibrosis and uninfected mice (n = 8) were intragastric administrations of equal value of carboxymethyl cellulose carrier. When animals were sacrificed at the last time of treatment, liver portal venous pressures were measured, and tissues (livers and spleens) and serum were collected. Perfusions of hepatic portal system were performed to detect the number of adult worms as described [22]. For *in vivo* experiments on primary mouse HSCs, the mice getting the infection for 12 weeks and then administrating 250 mg/kg PZQ for 3 days were employed (n = 6 in each group).

### Sirius Red staining and measure of liver hydroxyproline content

Mice livers were fixed in 10% neutralized formaldehyde and embedded in paraffin, and tissue sections (4-µm thick) were stained with aqueous saturated solution of picric acid containing 0.1% Sirius Red (Sigma-Aldrich, St. Louis, MO). Images of six random microscopic fields of red- stained collagen fibers in the liver section of each mouse were recorded using an inverted microscope (ZEISS, Goettingen, Germany) and then digitized and analyzed on Image-Pro Plus software 6.0 as previously described [21]. The content of liver hydroxyproline was determined according to the protocol of the Hydroxyproline Testing Kit (Jiancheng, Nanjing, China) as described [23].

### Immunohistochemistry assay for α-SMA of liver

Smooth muscle actin alpha (α-SMA) (Sigma-Aldrich, St. Louis, MO), a marker of activated HSCs, was detected on formaldehyde-fixed paraffin-embedded sections by immunohisto- chemistry method. Images of six random microscopic fields in the sections of each mouse were recorded using an inverted microscope (ZEISS, Goettingen, Germany) and the α-SMA positive area of each image was analyzed by Image-Pro Plus software 6.0 software [21].

### Measurement of mice serum alanine aminotransferase (ALT) and aspartate aminotransferase(AST)

Mice blood were collected in 1.5 ml Eppendorf tubes and standing in room temperature for 4 hours. The serums were separated by centrifugation at 3500 r/min for 15 minutes and serum ALT/AST was detected by Olympus AU5400 automatic biochemistry analyzer.

### Measurement of liver portal venous pressure

Mouse was anesthetized with 2% Pentobarbital Sodium, then peritoneal cavity was opened and hepatic portal vein was canulated. Strain gauge transducer was used to get the signal from liver portal vein and the signal was recorded by PowerLab8SP (ADInstruments, Castle Hill, Australia). Data acquisition was performed using Chart software v5.4 [24].

### Isolation and culture of mice primary HSCs

HSCs were isolated from mice by the modified mothed as described [25]. Mice were anesthetized with 2% Pentobarbital Sodium. Peritoneal cavity was opened and hepatic portal vein was canulated. In situ perfusion of the livers was initiated with 40 mL DMEM (Hyclone, Thermo Fisher Scientific, Beijing, China) and at the same time the suprahepatic inferior vena cava was cut. The liver was removed, placed in a dish and perfused continually with DMED containing 0.04% collagenase type IV and 0.2% pronase (Gibco Life Technologies, Grand Island, NY, USA) at 37°C for 10 minutes. Then, the liver was further digested with DMEM containing 0.08% collagenase type IV, 0.08% pronase and 10 U/ml DNase I (Sigma-Aldrich, St. Louis, MO) at 37°C bath shaking for 30 minutes. The resulting cell suspension was filtered through a nylon mesh and washed with cold DMEM for three times. Ten percent Optiprep(Axis-Shield PoC AS, Oslo, Norway) was used for density gradient centrifugation to isolate HSCs [26], [27] and purity of HSCs was estimated based on the autofluorescence of the cells by ultraviolet-excited fluorescence microscopy. Cell viability was examined by Trypan blue exclusion. Both cell purity and viability were in excess of 90% [28].

### RNA isolation and Real Time PCR

Tissues or cells were homogenized in Trizol (Invitrogen, Carlsbad, CA) and their RNAs were extracted according to the manufacturer's protocol. The RNA purity was assessed by spectrophotometry and 2% (w/v) agarose gel electrophoresis. Reverse transcriptase (RT) reactions for cDNA synthesis were carried out using RevertAid™ First Strand cDNA Synthesis Kit with oligo-dT primer (Fermentas,EU). Relative expression of mRNA species was determined by Real Time PCR with Faststart Universal SYBR Green PCR Master(Roche Diagnostics, Indianapolis, IN, USA) using ABI7300. The primers formouse GAPDH (Forward 5′-TGGAAAGCTGTGGCGTGAT-3′, Reverse 5′-TGCTTCACCACCTTCTTGAT-3′) [29], mouse TGF-β (Forward 5′-CACCGGAGAGCCCTGGATA-3′, Reverse 5′-TGTACAGCTGCCGCACACA-3′) [30],mouse α-SMA(Forward 5′-TCAGCGCCTCCAGTTCCT-3′, Reverse 5′-AAAAAAAACCACGAGTAACAAATCAA-3′) [30],mouse Col1α1(Forward 5′-ACGTCCTGGTGAAGTTGGTC-3′, Reverse 5′-CAGGGAAGCCTCTTTCTCCT-3′) [31],mouse Col3α1(Forward 5′-TGGTCCTCAGGGTGTAAAGG-3′, Reverse 5′-GTCCAGCATCACCTTTTGGT-3′) [32],mouse MMP9(Forward 5′-GCTCATGTACCCGCTGTATAGCT-3′, Reverse 5′-CAGATACTGGATGCCGTCTATGTC-3′) [33], mouse TIMP1(Forward 5′-TGGGAAATGCCGCAGATATC-3′, Reverse 5′-TGGGACTTGTGGGCATATCC-3′) [33], mouse IL-4(Forward 5′-GGTCTCAACCCCCAGCTAGT-3′, Reverse 5′-GCCGATGATCTCTCTCAAGTGAT-3′), mouse IL-10(Forward 5′-GCTCTTACTGACTGGCATGAG-3′, Reverse 5′-CGCAGCTCTAGGAGCATGTG-3′), mouse IL-13(Forward 5′-CCTGGCTCTTGCTTGCCTT-3′, Reverse 5′-GGTCTTGTGTGATGTTGCTCA-3′) and mouse IFN-γ(Forward 5′-ATGAACGCTACACACTGCATC-3′, Reverse 5′-CCATCCTTTTGCCAGTTCCTC-3′)were gained from articles orPrimerBank (http://pga.mgh.harvard.edu/primerbank/), and used for PCR. Cycling conditions were 2 min at 50°C, then 10 min at 95°C and followed by 40 cycles of 95°C for 15 s and 60°C for 1 min. Melt curve analysis and 2% agarose gel electrophoresis of the amplicon were used to determine the specificity of the amplicon. Data were normalized with GAPDH and results were expressed as fold amplification using the method described by Pfaffl [34]. Each experiment was repeated three times.

### Statistics

The data were analyzed using two-tailed Student's *t*-test when compared between two groups. A p-value<0.05 was considered statistically significant. All statistical analyses were operated by GraphPad Prism software 5.0.

## Results

### PZQ Treatment Decreases Liver Fibrosis and Its Related Gene Expressions in the Mice with Chronic Schistosomiasis

**Figure 1:**
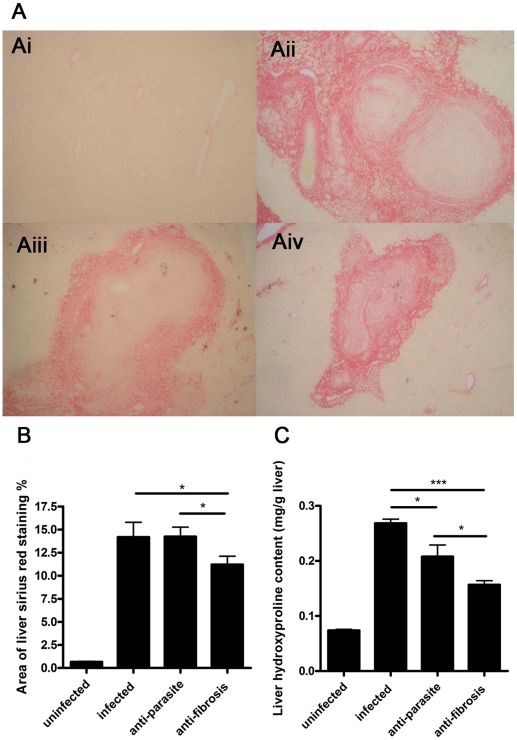
Anti-fibrosis treatment with prizaquantel reduced areas of liver Sirius Red staining and liver hydroxyproline contents of mice 8 weeks post infection with *Schistosoma japonicum*. (A) Representative images of Sirius Red staining by light microscope (×100). Ai, uninfected; Aii, infected; Aiii, anti-parasite; Aiv, anti-fibrosis. (B) Statistical analysis of scanned images in Sirius Red staining by software IPP6.0. (C) Measurement of liver hydroxyproline content. (* p<0.05, ** p<0.01, *** p<0.001).

**Figure 2:**
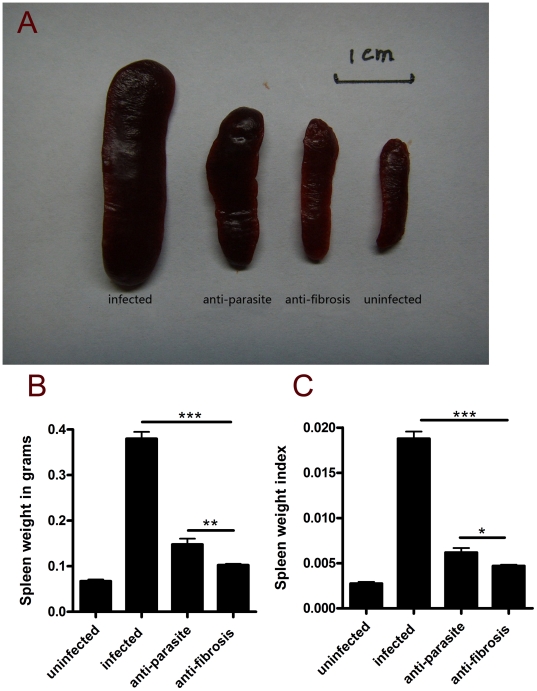
Anti-fibrosis treatment with prizaquantel reduced spleen weights in mice 8 weeks post infection with *Schistosoma japonicum*. (A) images of representative spleens in different groups. The bar is 1 cm. (B) Statistical analysis of spleen weights. (C) Spleen weight index (spleen weight/body weight). (* p<0.05, ** p<0.01, *** p<0.001).

**Figure 3:**
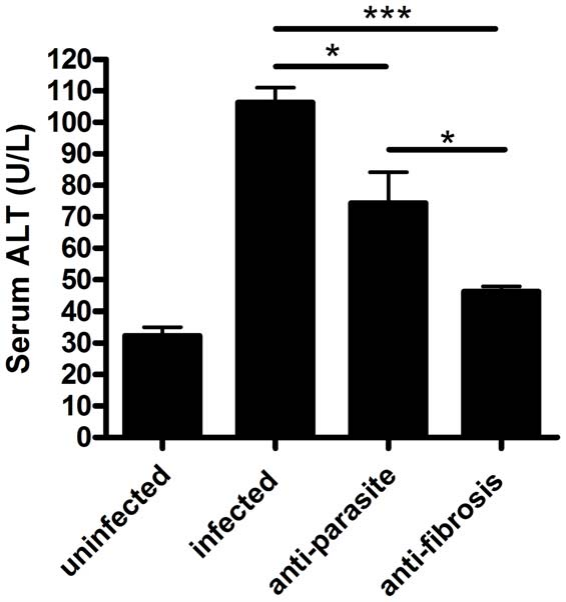
Anti-fibrosis treatment with prizaquantel reduced serum alanine aminotransferase (ALT) levels in mice 8 weeks post infection with *Schistosoma japonicum*. Serum ALT was assessed by Olympus AU5400 automatic biochemistry analyzer. (* p<0.05, ** p<0.01, *** p<0.001).

PZQ was administrated to the mice infected with *S. japonicum* at week 8 post-infection for either 3 days (anti-parasite group, 250 mg/kg/day) or 30 days (anti-fibrosis group,300 mg/kg/12 hours). Homochronous normal mice (uninfected group) and untreated mice (infected group) were also used in the experiments. The perfusion of hepatic portal system showed that no adult worm was detected in anti-parasite group at the end of the treatment (data not shown), which demonstrated that the administration of PZQ for anti-parasite treatment resulted in parasitologic cure. As shown in figures 1A and 1B, the areas of Sirius Red- stained liver were significantly decreased in anti-fibrosis group in comparison to both infected group and anti-parasite group. The accordant results were observed in measurement of liver hydroxyproline contents (Figure 1C). In addition, the spleen weight and the spleen index (spleen weight/body weight) of the mice were also reduced in anti-fibrosis group in comparison to both infected group and anti-parasite group (Figure 2). Serum ALT which was dramatically increased after the infection was decreased by anti-fibrosis therapy of PZQ (Figure 3).

**Figure 4:**
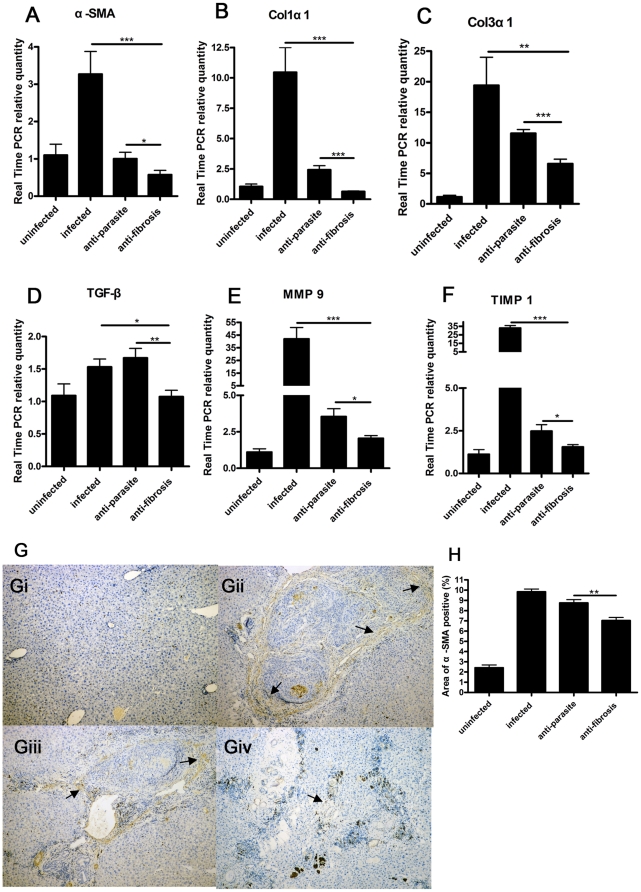
Anti-fibrosis treatment with prizaquantel inhibited expressions of liver profibrotic genes and α-SMA protein expression in mice 8 weeks post infection with *Schistosoma japonicum*. The expressions of mRNA were detected by Real Time PCR. (A) Expression of α-SMA. (B) Expression of Col1α1. (C) Expression of Col3α1. (D) Expression of TGF-β. (E) Expression of MMP9. (F) Expression of TIMP1. α-SMA protein expression was assessed by immunohistochemistry (G) Representative images of immunohistochemistry by light microscope (×100). Gi, uninfected; Gii, infected; Giii, anti-parasite; Giv, anti-fibrosis. Arrows showed the positive staining. (H) α-SMA positive areas were measured by software IPP6.0. (* p<0.05, ** p<0.01, *** p<0.001).

Hepatic mRNA expressions of the genes associated with liver fibrosis were detected by Real Time PCR. Results showed that the levels of Col1α1, Col3α1, α-SMA, MMP9 and TIMP1 were decreased while TGF-β was increased in anti-parasite group in comparison to infected group and all of them were decreased in anti-fibrosis group in comparison to both infected group and anti-parasite group (Figure 4A–F). Immunohistochemistry assay showed that α-SMA positive cells around the hepatic egg granulomas were dramatically decreased in anti-fibrosis group in comparison to both infected group and anti-parasite group (Figure 4G–H).

Besides, CCL_4_-induced early and established liver fibrosis mice [35] was brought into our experiment (Text S1). PZQ treatment significantly reduced the collagen areas in CCL_4_-induced fibrotic liver assessed by Sirius Red staining (Figure S1A–D). Content of hepatic hydroxyproline (Figure S1E) as well as serum ALT and AST (Figure S1F) was also decreased significantly in PZQ treated mice.

### PZQ Treatment Decreases Liver Fibrosis and Enhances MMP9 gene Expression of Mice with Advanced Schistosomiasis

**Figure 5:**
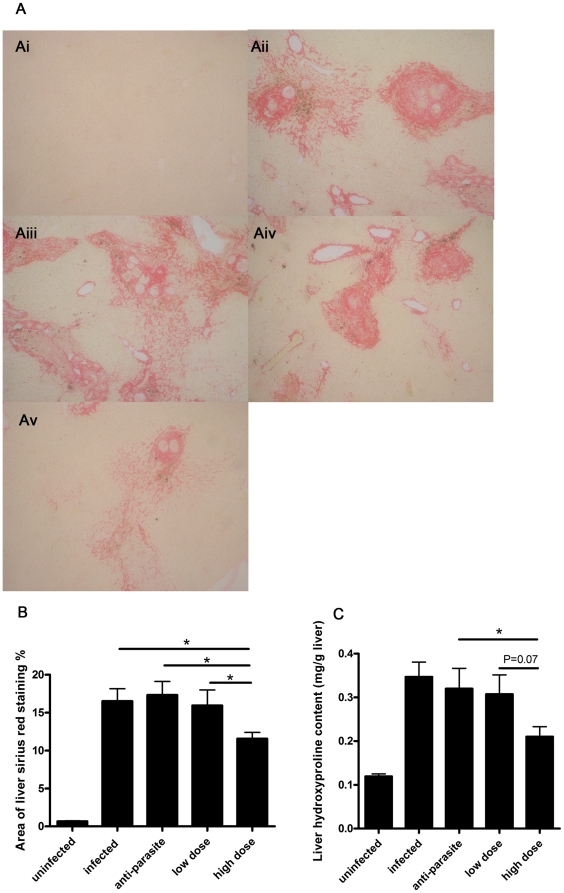
Anti-fibrosis treatment with prizaquantel reduced areas of liver Sirius Red staining and liver hydroxyproline content in mice 15 weeks post infection with *Schistosoma japonicum*. (A) Representative images of Sirius Red staining by light microscope (×100). Ai, uninfected; Aii, infected; Aiii, anti-parasite; Aiv, low dose; Av, high dose. (B) Statistical analysis of scanned images in Sirius Red staining by software IPP6.0. (C) Measurement of Liver hydroxyproline content. (* p<0.05).

**Figure 6:**
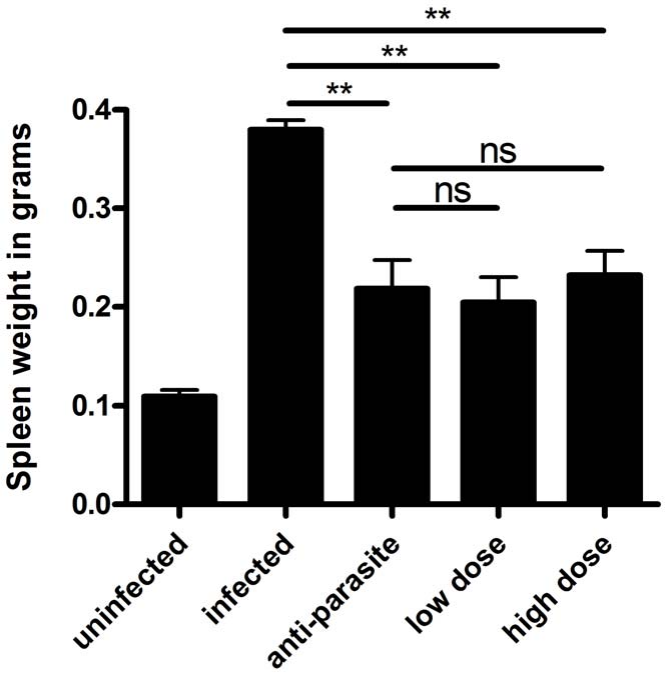
Spleen weights were not changed after Anti-fibrosis treatment with low dose or high dose prizaquantel compared with anti-parasite treatment in mice 15 weeks post infection with *Schistosoma japonicum*. Spleen weights were reduced in anti-parasite, anti-fibrosis low dose and high dose groups compared with infected group, but no significant changes were found between treated groups. (** p<0.01).

**Figure 7:**
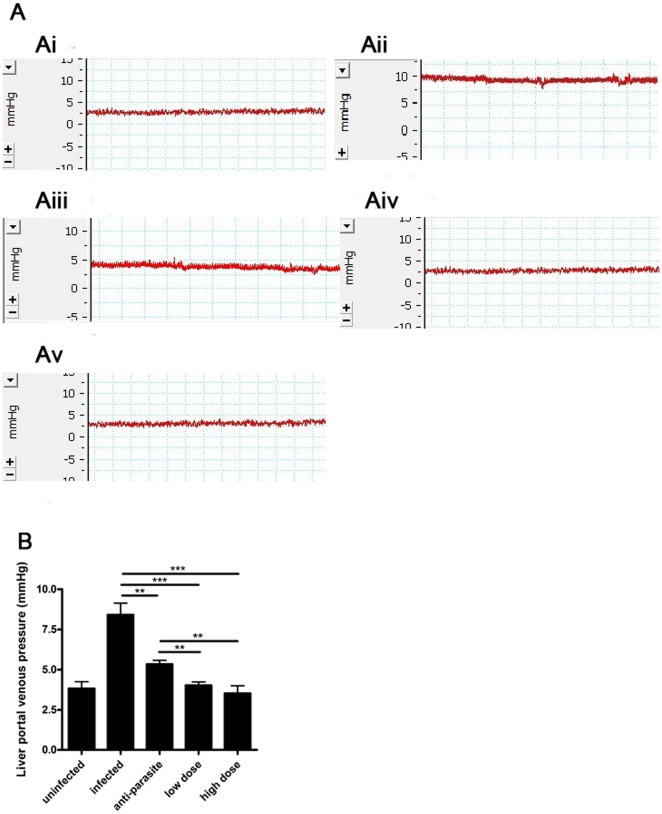
Anti-fibrosis treatment with prizaquantel reduced hepatic portal venous pressure. Hepatic portal venous pressure was measured by PowerLab8SP and analyzed by chart5 software. (A) Representative recording images were showed: Ai, uninfected; Aii, infected; Aiii anti-parasite; Aiv, low dose; Av, high dose. (B) Statistical analysis of each group hepatic portal venous pressure. Although anti-parasite treatment reduced portal venous pressure compared with infected group, anti-fibrosis treatment (both low dose and high dose group) further reduced the pressure to nearly uninfected level. (** p<0.01, *** p<0.001).

**Figure 8:**
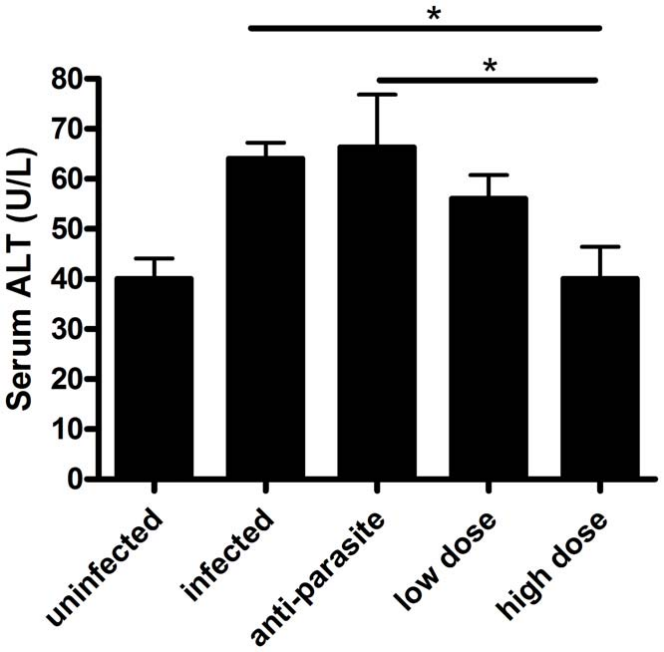
Anti-fibrosis treatment with prizaquantel reduced serum alanine aminotransferase (ALT) level in mice 15 weeks post infection with *Schistosoma japonicum*. Serum ALT was assessed by Olympus AU5400 automatic biochemistry analyzer. Levels of serum ALT were significantly decreased nearly to the normal level after anti-fibrosis treatment with high dose prizaquantel. (* p<0.05).

In this study, PZQ was administrated to the mice infected with *S.japonicum* at week 15 post-infection for either 3 days (anti-parasite group, 250 mg/kg/day) or 30 days (anti-fibrosis low dose group, 75 mg/kg/12 hours; anti-fibrosis high dose group, 300 mg/kg/12 hours). Homochronous normal mice (uninfected group) and untreated infected mice (infected group) were also used in the experiments. As expected, the areas of Sirius Red staining on the liver sections were significantly decreased in high dose group in comparison to both infected group and anti-parasite group. In low dose group, the Sirius Red stained-areas decreased without statistical significance in comparison to anti-parasite group. Also, the change profiles of the liver hydroxyproline contents were in accordance with those of Sirius Red staining (Figure 5). The spleen weights of mice in anti-parasite group and anti-fibrosis groups (both low dose and high dose) were decreased in comparison to infected group, but no significant differences between them (Figure 6). Portal hypertension is a characteristic manifestation of advanced schistosomiasis japonica and resulted mainly from severe hepatic fibrosis. To evaluate the anti-fibrosis effects of PZQ on the mice with advanced schistosomiasis, we measured liver portal venous pressure. The results showed that the pressure of the mice were significantly decreased after anti-parasite treatment and further decreased to nearly normal levels after both low dose and high dose treatment of PZQ (Figure 7). Serum ALT levels were significantly decreased only in high dose group, which were nearly back to normal baselines (Figure 8).

**Figure 9:**
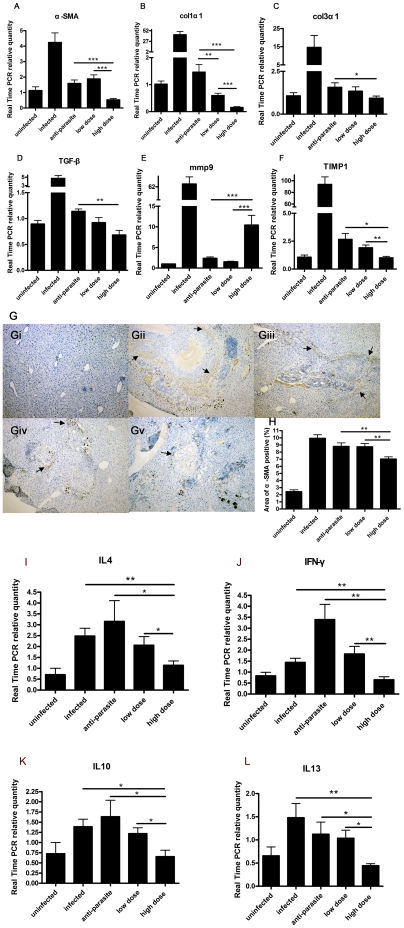
Anti-fibrosis treatment with prizaquantel inhibited expressions of profibrotic genes and α-SMA protein expression in liver, but increased MMP9 mRNA expressions of mice 15 weeks post infection with *Schistosoma japonicum*. (A)α-SMA; (B)Col1α1; (C)Col3α1; (D)TGF-β were reduced by anti-fibrosis treatment with high dose prizaquantel. (E)MMP9 levels in high dose group were increased about 10 fold compared with uninfected group, whereas (F) TIMP1 levels were decreased nearly to uninfected level. α-SMA protein expressions was assessed by immunohistochemistry. (G) Representative images of immunohistochemistry by light microscope (×100). Gi, uninfected; Gii, infected; Giii, anti-parasite; Giv, low dose; Gv, high dose. Arrows showed the positive staining. (H) α-SMA positive areas were measured by software IPP6.0. Expression of IL-4 (I), IL-10 (J), IL-13(K) and IFN-γ (L) were significantly reduced in high dose group compared respectively with infected, anti-parasite or low dose group. (* p<0.05, ** p<0.01, *** p<0.001).

**Figure 10:**
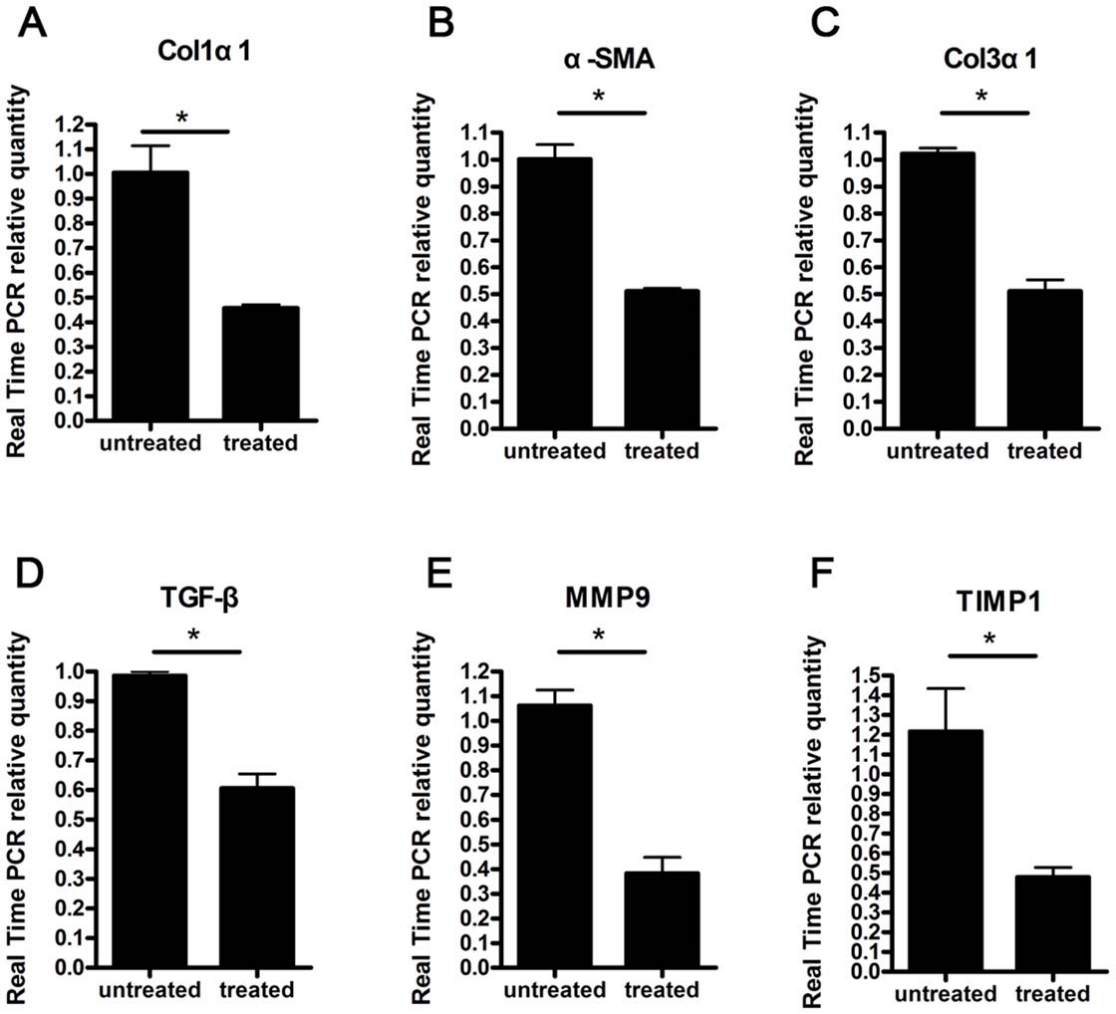
Treatment of prizaquantel for 3 days inhibited expressions of HSC profibrotic genes of mice 12 weeks post infection with *Schistosoma japonicum*. Prizaquantel (250 mg/kg/day) was administrated to mice for 3 days. Primary HSCs were isolated and transcriptional levels were assessed by Real Time PCR. Transcriptional level of (A) Col1α1, (B) α-SMA,(C) Col3α1, (D) TGF-β, (E) MMP9 and (F) TIMP1 in HSC of treated mice were significantly reduced compared with untreated mice. (* p<0.05).

We also detected the expressions of fibrosis-related genes. Col1α1, Col3α1, α-SMA and TGF-β mRNAs of liver were all markedly decreased in high dose group in comparison to both infected group and anti-parasite group (Figure 9A–D), which were similar to those changes in the mice with chronic schistosomiasis. Liver TIMP1 mRNA was reduced in low dose group, but more significantly decreased in high dose group in comparison to anti-parasite group. However, the expression of liver MMP9 mRNA was increased nearly five-fold in high dose group in comparison to both anti-parasite group and low dose group (Figure 10E–F). Hence, PZQ did not inhibit all kinds of fibrosis-related genes in liver. The immunohistochemistry change of liver α-SMA in each group was accordant with its α-SMA mRNA expression (Figure 9G–H). Gene Expressions of Th1/Th2 cytokines of IL4, IL10, IL13 and IFN-γ were all reduced in high dose group in comparison to infected, anti-parasite and low dose group, respectively (Figure 9I–L). Normal mice treated with high dose PZQ for 30 days (Text S2) showed reduced mRNA expression of Col1α1, TGF-β and TIMP1 in liver in comparison to untreated control mice (Figure S2A, D and F). Col3α1 and α-SMA (Figure S2B and C) showed a tendency to decrease, while MMP9 (Figure S2E) showed a tendency to increase, but the changes were not significant statistically. We also demonstrated that PZQ did not inhibit the proliferation of splenic mononuclear cells stimulated by schistosomal egg antigens detected by incorporation of ^3^H-thymidine (TDR) (Text S3 and Figure S3).

### PZQ Treatment Inhibits Activation or Fibrosis-related gene expressions of Primary HSCs in Mice with Chronic Schistosomiasis

PZQ was administrated to the mice infected with *S.japonicum* at week 12 post-infection for 3 days (250 mg/kg/day) and primary HSCs were isolated for detection of mRNA expression. Consistent with anti-fibrosis treatment experiment of the mice with chronic schistosomiasis, the expressions of activation or fibrosis-related genes such as Col1α1, Col3α1, α-SMA, TGF-β, MMP9 and TIMP1 were all decreased significantly after the anti-parasite treatment with PZQ (Figure 10).

## Discussion

Although it remain a neglected transmissible disease, affecting people mainly in developing countries, schistosomiasis are second only to malaria as a major parasitic disease [36]. So far, although chemotherapy makes a great success in against all schistosome species, few admitted and widely used interventions of anti-fibrosis can be accepted to improve the hepatic fibrosis and treatment of hepatic fibrosis for the patients with schistosomiasis is neglected all the while. The patients with severe diseases have no choice but surgery for enlarged spleens or fibrotic liver to avoid the risk of death [37], [38]. Consequently, radical therapy of hepatic fibrosis remains an imperative requirement and new effective drugs of anti-fibrosis are eager to be applied in clinical.

PZQ, as an antihelminthic drug, is widely used now. After PZQ anti-parasite therapy, hepatic fibrosis is reversal in murine schistosomiasis model and the reversal is considered due to pathogenic cure [39]. Other effects have been reported too in recent years [19], [20], suggesting that PZQ may have direct effect on host. However, there is no report about the direct anti-fibrosis effect and mechanism of PZQ on liver. In our experiments, we adopted continuous and therapeutic dose (600 mg/kg/day for murine approximately converts to 60 mg/kg/day for human [40]) of PZQ for anti-fibrosis treatment and investigated whether it has the effect of anti-fibrosis and contributes to the strategy of schistosomiasis hepatic fibrosis treatment.

Because mouse model is of high homologization and has similar course with human schistosomiasis, we thus established the mouse schistosomiasis model [41]. In the study, we demonstrated that anti-fibrosis treatment with PZQ significantly improved the hepatic fibrosis of schistosomiasis japonica at both 8 weeks and 15 weeks post-infection, as measured by Sirius Red staining areas and hydroxyproline content. We also detected the clinical parameters, such as spleen weight, portal venous pressure and serum ALT. The results showed that clinical symptoms, such as splenomegaly [42], [43], portal hypertension [44], [45] and dysregulated liver function [46], [47] were ameliorated by anti-fibrosis treatment with PZQ. Low dose treatment with PZQ did not show significant effects on hepatic fibrosis, which may be due to the quick metabolism of this drug in liver [48]. Because of the probable formation of fibrotic spleens [47], the enlarged spleens were not reversed by treatment of PZQ in advanced schistosomiasis mice. Meanwhile, hepatic fibrosis was more serious in 15 weeks- post-infected mice than in 8 weeks- post-infected mice before and after treatment. Herein, we suggest that anti-fibrosis treatment with PZQ should be used as early as possible and delayed treatment will be difficult to reverse the splenomegaly and hepatic fibrosis.

To further confirm the reversal of hepatic fibrosis, we detected the transcriptional level of some profibrotic genes in liver, such as Col1α1 and Col1α3 which are predominant collagens in the liver of schistosomiasis mice [49], [50]. TGF-β is considered as typical profibrotic gene in the progress of hepatic fibrosis [51], [52], and also plays an important role in hepatic fibrosis of schistosomiasis [53], [54]. The expressions of TGF-β, as well as α-SMA which is the activated marker of HSCs [7], [55], were detected between each group. All of them were decreased after anti-fibrosis treatment of PZQ, suggesting that anti-fibrosis treatment of PZQ inhibits transcriptional levels of profibrotic gene expressions in schistosomiasis mouse liver.

Besides, gene expressions of Th1/Th2 cytokines such as IL-4, IL-10, IL-13 and IFN-γ, which were important in schistosomiasis liver fibrosis [21], [56], [57], were also detected in our experiment. Studies in schistosomiasis demonstrated that the development of fibrosis requires the production of the profibrotic cytokines IL-4 and IL-13 [58], [59], [60]. Oppositely, IL-10 and IFN-γ showed antifibrotic effects [57]. Our results demonstrated that anti-fibrosis treatment with PZQ significantly inhibited the gene expression of both Th1 and Th2 cytokines, which suggested that the alteration of Th1/Th2 cytokines in hepatic microenvironment after PZQ treatment might finally influence the resolution of hepatic fibrosis. Furthermore, although expressions of fibrosis associated genes in liver of normal mice were low, PZQ treatment could also inhibit the expression of them such as Col1α1, TGF-β and TIMP1 in our experiment. Therefore, PZQ treatment could regulate the expression of fibrosis associated genes not only on schistosomiasis mice but also on normal mice.

The balance of the activities between matrix metalloproteinases (MMPs) and TIMPs(tissue inhibitors of metalloproteinases) plays an important role in extracellular matrix(ECM) production and degradation in hepatic fibrosis [61], [62]. MMP9 with the activity of collagen degradation [21], [63] has multi-source and is produced by Kupffer cells [64], hepatocytes [65] and HSCs [66]. Meanwhile, HSCs is also the main source of TIMP1 [8]. In our chronic fibrosis model, we showed that treatment of anti-fibrosis with PZQ reduced transcriptional levels of MMP9 and TIMP1. Meanwhile, TIMP1 was more close to the normal level than MMP-9. Unexpectedly, we observed a high transcriptional level of MMP9 by anti-fibrosis treatment with PZQ in liver of advanced infected mice, whereas TIMP1 was significantly reduced. Thus, the imbalance that favors MMP9 by PZQ treatment is helpful to reverse hepatic fibrosis.

Unlike schistosome egg induced hepatic granuloma and fibrosis, CCL_4_-induced liver fibrosis is mainly due to the damage of hepatocyte [35]. However, the mechanism of resulting liver fibrosis is the same that is the activation of HSCs and subsequent ECM deposition [5]. In order to confirm the anti-fibrotic properties of PZQ, we established mice model of CCL_4_-induced liver fibrosis. As expected, PZQ treatment significantly reduced liver collagen areas and hydroxyproline content in mice of CCL_4_-induced liver fibrosis. Level of serum ALT and AST were also significantly reduced after PZQ treatment. These results further confirmed the anti-fibrosis effects of PZQ.

To further investigate cellular mechanism of anti-fibrosis with PZQ, we focused on HSCs, which is one of liver resident cells and plays the key role in liver fibrosis [5], [7]. In recent researches, HSCs is the promising target of anti-fibrosis drugs [67]. In our experiments with mouse models, we found the reduced transcriptional levels of the fibrosis-related genes Col1α1, Col3α1, α-SMA, TGF-β, MMP9 and TIMP1 in primary HSCs by PZQ treatment for 3 days. This result was consistent with anti-fibrosis treatment experiment of mice with chronic schistosomiasis, indicating that inhibition of HSCs activation by PZQ occured rapidly after the treatment. More importantly, the inhibition was persistence and no rebounding of this effect was observed at the end of anti-parasite treatment. Therefore, PZQ might exhibit the anti-fibrosis property by affecting activation of HSCs. Although the appropriate dose-course of anti-fibrosis treatment by PZQ is not determined and the exact pathway is not clear, our results suggest that PZQ is a promising drug for the treatment of hepatic fibrosis.

In conclusion, although the pathways about anti-fibrosis property of PZQ are enigmatic, we demonstrated the effects of anti-fibrosis of PZQ in mice with both chronic and advanced schistosomiasis as well as in CCL_4_-induced liver fibrosis mice. Our study also provided clues that based on the changes following PZQ treatment, the levels of TGF-β and TIMP-1 were potential biomarkers for regression of liver fibrosis. It is suggested that new treatment strategy in clinical is that treatment of pathogenic intervention could be adopted at first and followed by anti-fibrosis treatment with PZQ for several times. The inhibition of transcriptional levels of profibrotic genes in HSCs by treatment with PZQ is probably part mechanism of its anti-fibrosis property. Further molecular mechanism and clinical application of PZQ are deserved to be investigated.

## Supporting Information

Figure S1 **PZQ treatment improved CCL_4_ -induced liver fibrosis and serum transaminase.** Liver sections of mice from normal (A), CCL_4_ -induced liver fibrosis (B), and PZQ treatment (C) groups were stained with Sirius Red, respectively. Statistical analysis showed that PZQ treatment significantly decreased collagen areas (D). PZQ treatment also decreased the liver hydroxyproline content (E) and serum ALT/AST (F). (*, p<0.05;**, p<0.01; ***, p<0.001).
(TIF)

Figure S2 **PZQ treatment inhibited the expressions of liver fibrosis associated genes in normal mice detected by Real Time PCR.** Results showed that PZQ treatment significantly decreased the expression of Col1α1 (A), TGF-β (D) and TIMP1 (F) (p<0.001), and the changes of Col3α1(B), α-SMA(C), and MMP9(E) were not significant. (p>0.05).
(TIF)

Figure S3 **Proliferation of spleen mononuclearcells were not significantly changed by stimulation of SEA.** 12 weeks post-infected schistosomiasis mice were first treated by prizaquantel or control solution. Then spleen mononuclearcells were isolated and stimulated by SEA or anti-CD3. The method of incorporation of ^3^H-thymidine (TDR) was used to assess the proliferation. (ns, no significance).
(TIF)

Text S1 (DOC)

Text S2 (DOC)

Text S3 (DOC)
