# Supplementary figures and images for: New Insight into the Antifibrotic Effects of Praziquantel on Mice in Infection with Schistosoma japonicum
Source: PLoS One. 2011 May 24;6(5):e20247. doi: 10.1371/journal.pone.0020247 (PMC3101229; doi:10.1371/journal.pone.0020247)

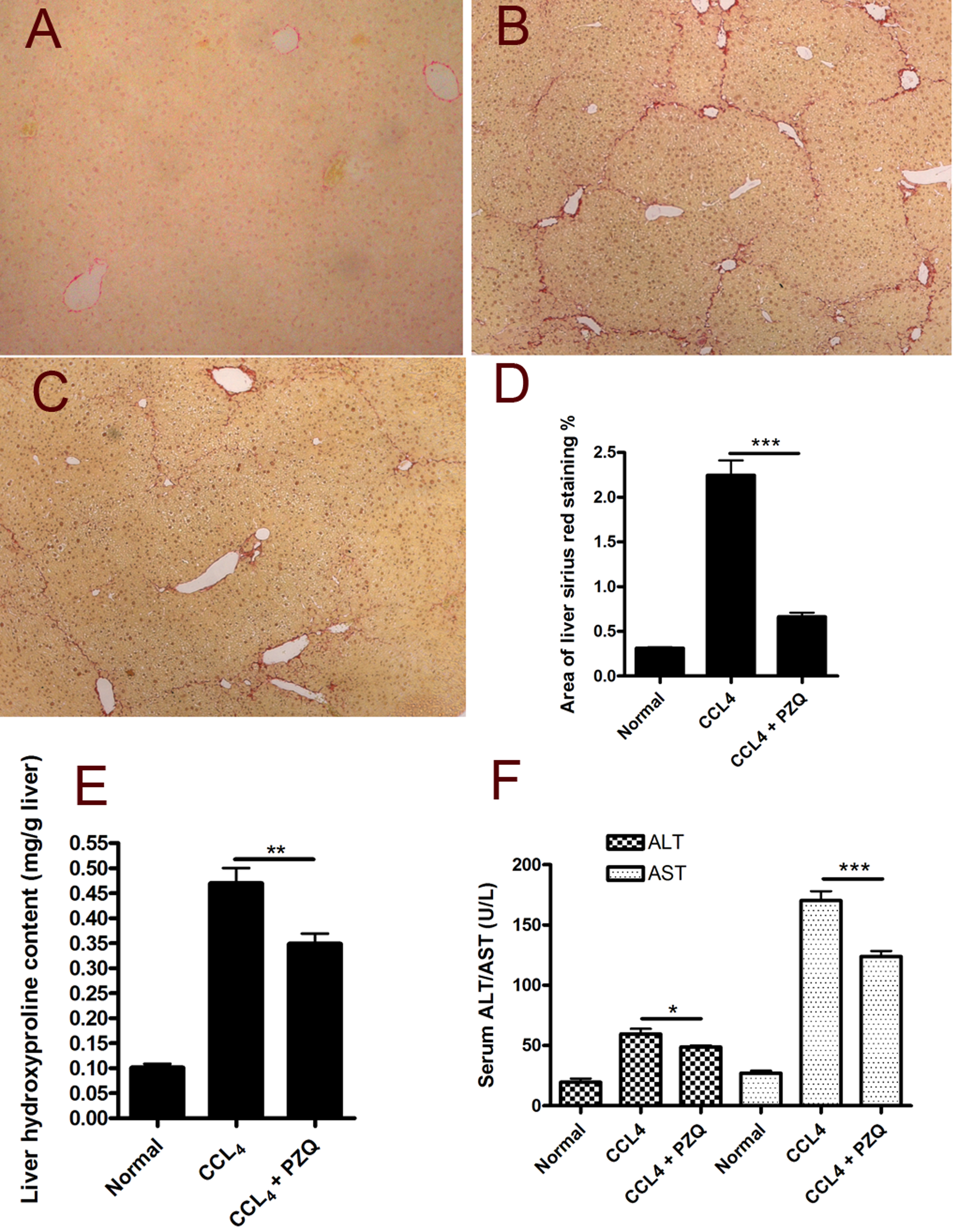

Supplement: Figure S1 — PZQ treatment improved CCL4 -induced liver fibrosis and serum transaminase. Liver sections of mice from normal (A), CCL4 -induced liver fibrosis (B), and PZQ treatment (C) groups were stained with Sirius Red, respectively. Statistical analysis showed that PZQ treatment significantly decreased collagen areas (D). PZQ treatment also decreased the liver hydroxyproline content (E) and serum ALT/AST (F). (*, p<0.05;**, p<0.01; ***, p<0.001). (TIF) [file pone.0020247.s001.tif]

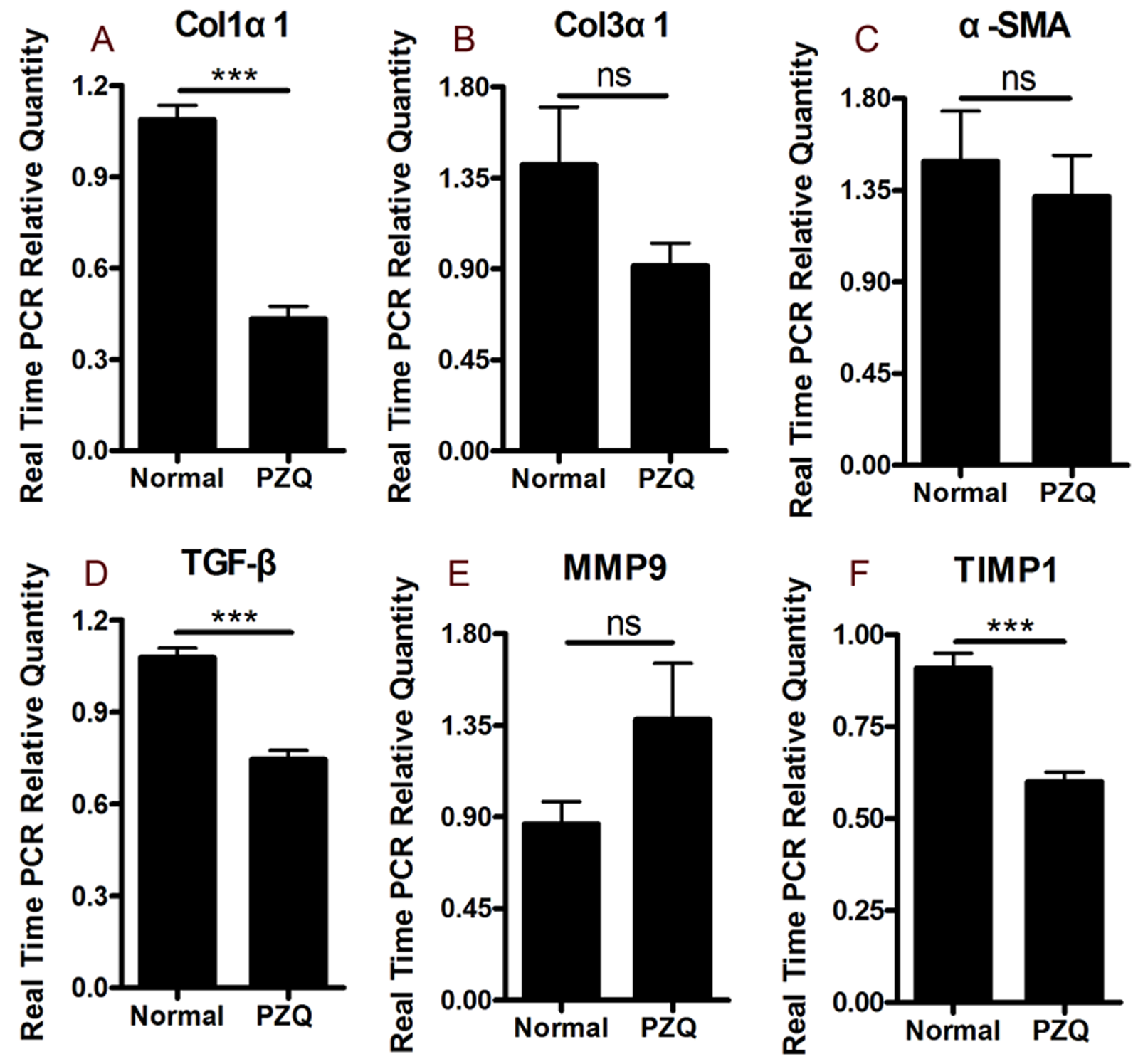

Supplement: Figure S2 — PZQ treatment inhibited the expressions of liver fibrosis associated genes in normal mice detected by Real Time PCR. Results showed that PZQ treatment significantly decreased the expression of Col1α1 (A), TGF-β (D) and TIMP1 (F) (p<0.001), and the changes of Col3α1(B), α-SMA(C), and MMP9(E) were not significant. (p>0.05). (TIF) [file pone.0020247.s002.tif]

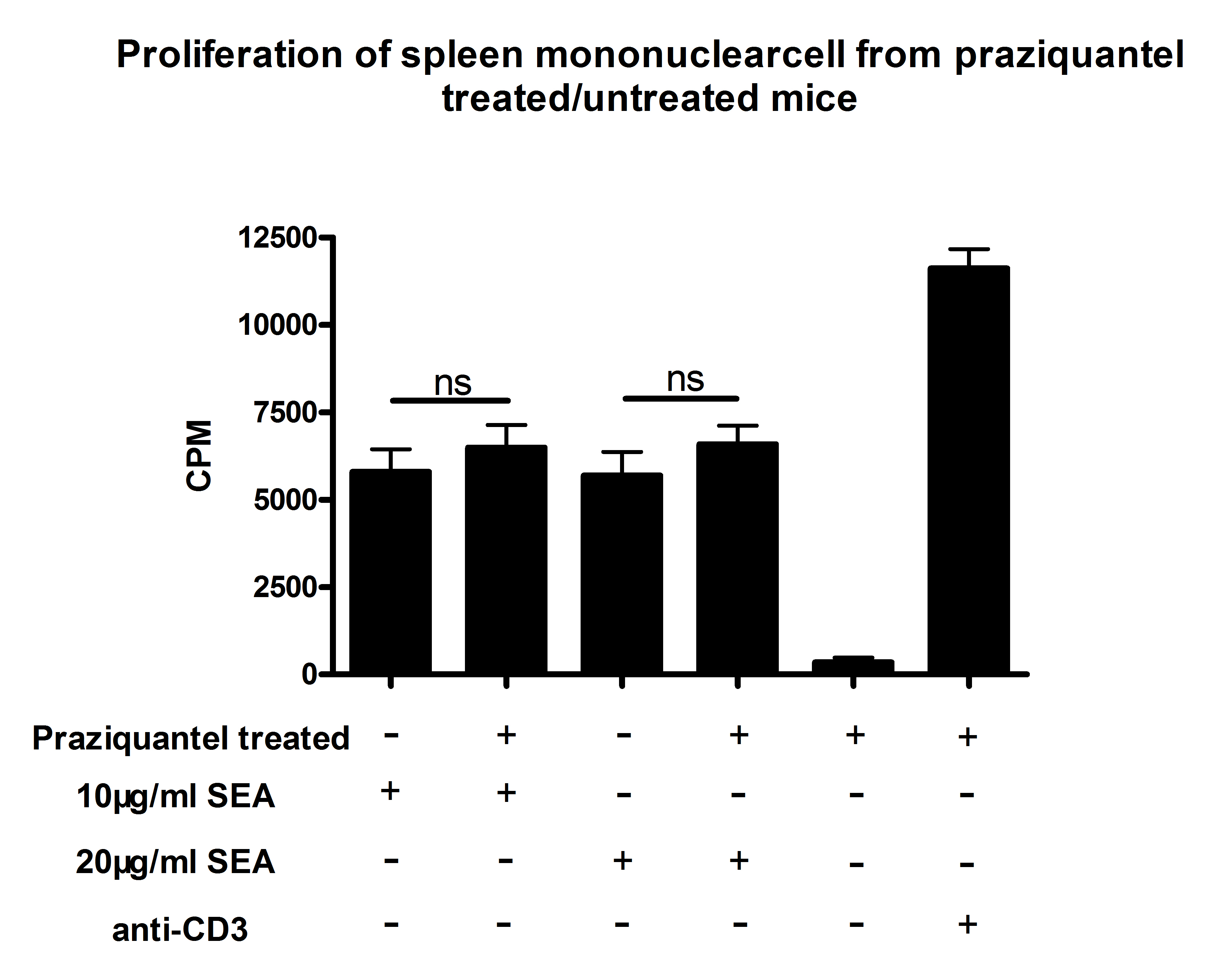

Supplement: Figure S3 — Proliferation of spleen mononuclearcells were not significantly changed by stimulation of SEA. 12 weeks post-infected schistosomiasis mice were first treated by prizaquantel or control solution. Then spleen mononuclearcells were isolated and stimulated by SEA or anti-CD3. The method of incorporation of 3H-thymidine (TDR) was used to assess the proliferation. (ns, no significance). (TIF) [file pone.0020247.s003.tif]
