# Supplementary material for: New Insight into the Antifibrotic Effects of Praziquantel on Mice in Infection with Schistosoma japonicum
Source: PLoS One. 2011 May 24;6(5):e20247. doi: 10.1371/journal.pone.0020247 (PMC3101229; doi:10.1371/journal.pone.0020247)
Supplement: Text S1 — (DOC) [file pone.0020247.s004.doc]

**Method**

Female BABL/c mice, 6-8 weeks old, were treated twice a week with CCl_4_ at 1 ml/kg in olive oil for 4 weeks to develop early and established fibrosis. Administration of PZQ (300mg/kg/12 hours) was used for 4 weeks and 1% Carboxymethyl Cellulose was used as control. Each group contained 10 mice. Mice were sacrificed at the end of the treatment. Sirius Red staining and hydroxyproline measurement of lives were performed. ALT/AST of serum was also detected.

**Result**

As shown in Figure S1, collagen areas as well as hydroxyproline content of PZQ treatment group (CCL_4_+PZQ) were significantly decreased compared with CCL_4_ –induced liver fibrosis group (CCL_4_) (**,P<0.01;***,P<0.001). Both ALT and AST were significantly decreased in PZQ treatment group (CCL_4_+PZQ) compared with that of CCL_4_ –induced liver fibrosis group (CCL_4_) (*, P<0.05;***, P<0.001).
