# Supplementary material for: New Insight into the Antifibrotic Effects of Praziquantel on Mice in Infection with Schistosoma japonicum
Source: PLoS One. 2011 May 24;6(5):e20247. doi: 10.1371/journal.pone.0020247 (PMC3101229; doi:10.1371/journal.pone.0020247)
Supplement: Text S2 — (DOC) [file pone.0020247.s005.doc]

**Method**

**PZQ treatment for normal mice**

Female BABL/c mice, 6-8 weeks old, were divided into 2 groups. Each group contained 6 mice. PZQ group were administrated with PZQ (300mg/kg/12 hours) and normal group were administrated with 1% Carboxymethyl Cellulose as control. All mice were sacrificed after 30 days treatment and total RNA of livers were extracted for Real Time PCR.

**Result**

As shown in Figure S2, gene Expressions of Col1α1 (A), TGF-β (D) and TIMP1 (F) were decreased significantly in PZQ group in comparison to normal group (*p*<0.001), while that of Col3α1(B), α-SMA (C)and MMP9(E) were not changed significantly (*p*>0.05).
