# Supplementary material for: New Insight into the Antifibrotic Effects of Praziquantel on Mice in Infection with Schistosoma japonicum
Source: PLoS One. 2011 May 24;6(5):e20247. doi: 10.1371/journal.pone.0020247 (PMC3101229; doi:10.1371/journal.pone.0020247)
Supplement: Text S3 — (DOC) [file pone.0020247.s006.doc]

**Method**

**Proliferation assess of spleen mononuclear cells by incorporation of ^3^H-thymidine (TDR)**

12 weeks post-infected mice were treated with prizaquantel (250mg/kg/day) for 3 days. Carboxymethylcellulose sodium solution was given to 12 weeks post-infected mice for untreated control. Spleen mononuclear cells were isolated. Different concentrations of schistosome egg antigen (SEA, 10μg/ml and 20μg/ml) and positive stimulation (anti-CD3) were used for proliferation of cells for 72 hours. ^3^H-thymidine was added 16 hours before the assay.

**Result**

After prizaquantel treatment (250mg/kg/day, 3 days), the proliferation of spleen mononuclearcells were not significantly changed by SEA stimulation compared with that of untreated mice (Figure S3).
